# Life as an early career researcher: Ruth Bower

**DOI:** 10.4155/fsoa-2016-0045

**Published:** 2016-07-14

**Authors:** Ruth Bower

**Affiliations:** 1 School of Life Sciences, University of Hull, Cottingham Road, Hull, HU6 7RX, UK

**Keywords:** early career, head and neck cancer, microfluidics, radiotherapy, tissue culture

## Abstract

**Ruth Bower talks to Francesca Lake, Managing Editor:** Ruth is currently researching head and neck cancer chemoradiotherapy regimens utilizing microfluidic technology to maintain and interrogate biopsies. Tissue response is investigated using a variety of whole tissue and cellular analytical techniques with a view toward personalized medicine. She is currently pursuing her PhD within the head and neck cancer research group at Hull University (UK). Ruth obtained a first class (Hons) degree in Biological Sciences from Lancaster University (UK) during which time she spent a year at the University of Wollongong (Australia).

## Q Can you tell us a little about your career path to date?

I have been driven and motivated by science from an early age; while still in senior school, I completed a 4-week research project through the British Science Association. My career path began at Lancaster University (UK) where I undertook a BSc in Biological Sciences; as part of the program, I spent a year abroad at the University of Wollongong (Australia), which was a great pleasure and challenge in almost equal measure. It was during my first longer-term research project at Lancaster University, on the addition of cannabidiol to standard chemotherapeutics to treat Leukemia cells, that my ‘fate’ was sealed. The idea (and reality, sometimes) that I could walk out of the laboratory at the end of the day having experimentally determined something I did not know at the start of the aforementioned day was, and still is, really exciting. It is also from that project that my research interests in oncology were cemented, which led me to undertake a PhD at the University of Hull (UK) within the head and neck cancer group.

## Q What are you working on at the moment?

I am currently working on the development of a system to enable personalized treatment for solid tumors. A bespoke tumor-on-chip device is employed to maintain patient tumors taken at resection in a pseudo *in vivo* environment. Precision-cut tumor slices are maintained under continuous flow within the device and are exposed to preoptimized radio and chemoradiotherapy regimens. Tissue recovered from the device is then subjected to a wide series of analyses to gain an understanding of tissue response including cell viability and death, proliferation and intracellular signaling. Effluent from the system is also utilized for the study of secreted soluble markers. The concept of the technology is that following a solid tumor diagnosis a biopsy could be taken and maintained in parallel devices for investigation of treatment options (standard chemotherapy/radiotherapy/hormone and emerging therapies or combinations thereof) with the most successful treatment regimen for the individuals’ specific tumor then being administered on the patient.

## Q What do you enjoy most about your work, and what do you find most challenging?

Fundamentally, I enjoy being in the laboratory, solving problems and obtaining results, particularly when multiple analyses and methodologies come together to tell a coherent story. I am also a passionate communicator so I relish every opportunity to present my work both in a conference or seminar setting and in public engagement festivals or activities. My current project is multidisciplinary in nature meaning discussion and collaboration with scientists from all disciplines, engineers and clinicians. I really enjoy this aspect of my work as it pushes boundaries of my own knowledge and, as such, provides a great personal challenge alongside opportunity.

## Q What are you hoping to achieve in the next 10–20 years?

I hope that I make a marked difference in the acquisition of new knowledge, and for that knowledge to have a translational impact on patients. One of the great strengths of the team I work in currently is our close relationship with clinicians; we frequently consult with them in terms of research goals to ensure that the work will have impact in the clinic. This is a hallmark of my current research that I would hope to take into the next 10–20 years and beyond. Research is fascinating but if it stays in the laboratory, impact is limited. Ultimately, I would hope to find myself in a diverse job role that allows me to facilitate the above, while continuing to extend my current skill set.

## Q In your opinion, what would you say are the biggest challenges facing early career researchers?

This answer will be inherently biased given it is a challenge I will shortly navigate, but I would say the transition from ‘graduate student’ to ‘employee’. I would further suggest the above is true regardless of whether an individual is looking to industry or academia for their first doctoral-level appointment. In the former, graduate students often have little or no industry experience, which results in a challenge in gaining, and then integrating into a position of this nature. In the latter, funding is a long-standing issue that other ambassadors have covered in this series; additionally, in some cases, there is a skills gap in recent PhD graduates in terms of the knowledge of, and ability to write applications for, grants and fellowships. This leads to a ‘catch 22’ situation, whereby in order to obtain employment a grant needs to be written yet the skills to successfully write the aforementioned grant would be gained within the role.

## Q How would you suggest we tackle these issues?

I do not believe there is a one-size-fits-all solution and to a large extent I would place the responsibility on the individual to position themselves to gain the necessary skills for their intended path, for example, assisting in writing grant applications from an early stage. I also place huge weight on the development of communication and networking skills; every opportunity should be taken to provide these, and when provided utilized to the greatest extent possible by individuals. Taking a wider view some PhD programs now include a professional internship placement – I think this is a great initiative and an elegant way to develop a more well-rounded skill set. It would be great to see this rolled out more widely.

## Q What advice would you give someone who is considering getting their PhD?

Be sure you want one; it sounds obvious but some prospective PhD candidates are not clear on their research interests. If there is a research field that you are passionate about and will get you out of bed every morning for the next 3+ years then you are halfway there. Then, begin the search for research groups and PhD projects that align with that passion. Always send informal inquiries before a final application – you gain extra information and show an early interest: win, win. Also, read; look for publications from potential supervisors and their group – it sets you up really well for any potential interviews and gives a great insight into the work of the group. Extra points if your informal inquiry asks intelligent question(s) arising from one of the supervisor's papers.

## Q Finally, what is the most important thing you have learned from your career thus far?

I will give you three things: 1) We do not know more than we do know – and that is exciting; 2) Make patience your friend; 3) Believe in (but question) yourself, and be part of a team that can help you with that.

